# Practical Departments

**Published:** 1894-09-08

**Authors:** 


### PRACTICAL DEPARTMENTS.

### TOBACCO AND CIGARS.

During the late meeting of the British Medical Association at Bristol the members were afforded an object lesson in the manufacture of cigars and cigarettes and the preparation of tobacco of all kinds. Messrs. W. D. and H. O. Wills threw open their factories and warehouses for the inspection of the members, and those who availed themselves of the privileges were not only highly pleased with all they saw, but gained knowledge on many points which could not fail to be of interest and value to smokers. Messrs. Wills take a paternal interest in the well-being of their employés, who number upwards of a thousand hands. The bright faces of the workpeople and their healthy appearance were very striking. The firm has provided a restaurant, where the workpeople can obtain food of the best description at astonishingly low rates. We inspected these foods and the arrangements, and were much pleased with them. All who take an interest in the food supply of the poor might benefit by a visit to this department of Messrs. Wills' establishment.

CIGARS AND THEIR MANUFACTURE.

We have often seen cigar making in foreign factories, where the process is sometimes horrible and repugnant from the absence of cleanliness, both in the factories and amongst the workpeople. At Bristol cigar making has been reduced to a fine art; all the materials are of the best kind, and everything tends to remove the impression we have referred to. Not only are the workpeople neatly dressed, but there is a marked cleanliness and order in the factory which does away with all the objectionable features to be seen in foreign factories. Having carefully inspected the various tobaccos used, as they are received from abroad, when sorted, and after undergoing the various processes to which they have to be subjected in order to make the tobacco suitable for making up into the different brands for which Messrs. Wills are so deservedly famed, we have come to the conclusion, that smokers generally, and especially those people who smoke cigarettes and cigars, would be well advised to make a selection from the goods of this firm, and to stick to them. We have carefully tested nine brands of cigars made by Messrs. Wills, and treating them entirely on their merits, we are of opinion that very many of them surpass the great number of foreign brands which cost at least twice as much as the English-made cigars. In our view smokers, whether they be members of the medical profession or of the public, could not do better than procure boxes of the following brands: Labrador Infantines, Little Lord Fauntleroy (Regalia Britannica), and Diamante Rothschild's Regalia. These three brands represent various sizes of cigars of the very best tobacco and manufacture. They are excellently made, easy to smoke, wholesome, and most pleasant in flavour and aroma. Another brand, Lord Tennyson (Regalia Especial), should also be mentioned. All smokers will understand the advantage of smoking pure tobacco of the best manufacture, and our inspection convinces us that this is the only quality of goods which Messrs. Wills supply.

Cigarettes.

Cigarette making is carried to great perfection at Bristol. There the visitor sees the cigarette-making machine, with two miles of paper upon it being converted into cigarettes at the rate of 250 per minute. The cigarettes are of excellent quality, and the elegant way in which they are put up for the market makes them very attractive. The different styles of packing adopted are most ingenious, and enable a smoker to purchase ten of the best cigarettes for 6d. in a handy case, which preserves them from injury and lies flat in the pocket. The gold-tipped Sahara are especially good. The cigarettes are made from the best Bird's Eye, Navy Cut, Gold Flake, Three Castles, Honeydew, and each and all the brands of tobacco for which Messrs. W. D. and H. O. Wills are so well known.

Tobaccos.

We need say nothing in praise of the tobaccos of this firm. The Three Castles brand is one which we have smoked for years with complete satisfaction and comfort. Whatever cranks and sentimentalists may have to say against tobacco smoking, there can be no doubt that it tends to aid the hardest workers to produce the best work at a minimum of discomfort. The purity and excellent quality of the tobaccos supplied by Messrs. Wills makes smoking not only a pleasure but possibly even a duty—in some cases at any rate.

### RED RUBBER SHEETING.

We have received from Mr. Albert Browne, manufacturer and wholesale dealer in all kinds of indiarubber and vulcanite goods, &c., of Leicester, a sample of a "special Red Rubber Sheeting for hospital use," for which certain exceptional advantages are claimed, notably that it is more durable than the grey or drab sheeting in general use. It is equally antiseptic, bearing scrubbing and washing without any damage to the material, and the coat of rubber is particularly thick.

The sheeting is very pliable, and is warranted not to crack. Its price is by no means excessive, being 7s. per yard (36 inches wide). Any improvements in the way of economy and durability in this matter will be welcomed in the hospital world, for indiarubber sheeting constitutes a very considerable item of expenditure, and one very difficult of curtailment.

Mr. Browne has supplied the New Royal Infirmary, Derby, with this red sheeting, a fact which speaks for its excellence, and it has been invariably found to give satisfaction in other hospitals which have adopted its use. We may perhaps own to a preference for the colour of the grey or drab sheetings, to which we are so accustomed, but this is, of course, a mere matter of sentiment, and one which does not affect the main point to be considered in purchasing sheeting, namely that it shall be the most pliable and durable to be obtained.

